# Improving classification of mature microRNA by solving class imbalance problem

**DOI:** 10.1038/srep25941

**Published:** 2016-05-16

**Authors:** Ying Wang, Xiaoye Li, Bairui Tao

**Affiliations:** 1 Modern Educational Technology Center, Qiqihar University, No. 42, Wenhua Street, Qiqihar, Heilongjiang, 161006, China; 2 Institute of Biomedical Engineering, College of Automation, Harbin Engineering University, 145 Nantong Street, Nangang District, Harbin, Heilongjiang, 150001, China

## Abstract

MicroRNAs (miRNAs) are ~20–25 nucleotides non-coding RNAs, which regulated gene expression in the post-transcriptional level. The accurate rate of identifying the start sit of mature miRNA from a given pre-miRNA remains lower. It is noting that the mature miRNA prediction is a class-imbalanced problem which also leads to the unsatisfactory performance of these methods. We improved the prediction accuracy of classifier using balanced datasets and presented MatFind which is used for identifying 5′ mature miRNAs candidates from their pre-miRNA based on ensemble SVM classifiers with idea of adaboost. Firstly, the balanced-dataset was extract based on K-nearest neighbor algorithm. Secondly, the multiple SVM classifiers were trained in orderly using the balance datasets base on represented features. At last, all SVM classifiers were combined together to form the ensemble classifier. Our results on independent testing dataset show that the proposed method is more efficient than one without treating class imbalance problem. Moreover, MatFind achieves much higher classification accuracy than other three approaches. The ensemble SVM classifiers and balanced-datasets can solve the class-imbalanced problem, as well as improve performance of classifier for mature miRNA identification. MatFind is an accurate and fast method for 5′ mature miRNA identification.

MicroRNAs (abbreviated miRNA) are ~20–25 nucleotides, single stranded non-coding RNAs, which regulated gene expression by base pairing the 3′ untranslated region of mRNAs in the post-transcriptional level^1^. They play important roles in many biological process including cell division, and disease development^2^,^3^. In mammals, miRNAs have a typical processing steps as follows: primary miRNA transcript (pri-miRNA) is cleaved to miRNA precursor (pre-miRNA) by Drosha, and then pre-miRNA is processed into miRNA:miRNA* duplex approximately 22 nt long by the Dicer, afterward, one of the two strands of the newly formed duplex is correspond to a mature miRNA, and the other is degraded. In some case, both strands are functional^4^.

Within the last decade, for miRNA identification, great efforts have been devoted to identify the pre-miRNAs from pseudo pre-miRNAs which extract from protein coding regions and have similar stem-loop structures with genuine pre-miRNAs but have not been reported as pre-miRNAs, such as Triplet^5^, Mipred^6^, HeteroMirPred^7^, micropred^8^, PlantMiRNAPred^9^ ,mirnaDetect^10^ and the algorithm developed Huang *et al*.^11^,^12^,^13^. Recently, a series of up-to-data methods are proposed for pre-miRNA identification with a detailed research on feature selection and algorithm optimization, such as iMiRNA-PseDPC^14^, gapped kernels^15^, methods based on structure status^16^ and miRNA-dis^17^. And some powerful web servers have been proposed to extract the features from the RNA sequences, such as repRNA^18^, and Pse-in-One^19^. They solve problems of various kinds in pre-miRNA identification, and improve the performance of classification. The ratio of the positive dataset and negative dataset of these approaches are usually larger than 1:10. In order to improve classification performance with respect to imbalanced dataset including positive and negative dataset, some initial attempts have been made for class-imbalance problem of pre-miRNAs prediction. MicroPred^8^ inspected five algorithms for class imbalance problem including SMOTE, multiple classifier system (MCS), Different error costs (DEC) and zSVM, And the result indicated that the SMOTE algorithm was most efficient. HeterMirPred^7^ employed improved rebalance algorithm based on SMOTE bagging. At first, the method extracted the minority class by the radio of 50% using SMOTE. Secondly, it selected the majority class utilizing under-sampling technique to achieve data balance. Xuan Tho Dang^20^ proposed over-sampling algorithm which named incre-mental-SMOTE on basis of improved SMOTE. And it was superior to SMOTE and others improved SMOTE algorithms including safe-level-SMOTE and bor-der-line-SMOTE in terms of sensitivity and G-average. Although many computational approaches show excellent performance for predicting new pre-miRNAs, miRNAs involved in biological process through mature miRNAs, not pre-miRNAs. The accurate identification of mature miRNAs is important for further study on miRNAs identification.

For mature miRNAs identification, the computational method must identify the start nucleotide of true mature miRNAs from 60–70 nucleotides within their pre-miRNA sequence. Therefore, mature miRNA identification problem should be also considered as imbalance class distribution. Several computational methods which have been developed for mature miRNAs classification include MatureByes^21^, MiRMat^22^, MiRRim2^23^, MiRdup^24^, MaturePred^25^, MiRPara^26^, mirExplorer^27^, Matpred^28^ and MiRduplexSVM^29^. These methods mainly focus on the use of machine learning techniques, and their lower predictive performance suffers from the class-imbalance problem. Only a few methods propose measures to solve the problem of class imbalance problem in mature miRNAs prediction. MaturePred^25^ uses a two-stage sample selection algorithm to treat the class imbalance problem. At first, the negative samples distribution is conformed using κ-Nearest Neighbor (κ-NN). Secondly, the negative samples which lead to the largest deviation on the current prediction model are selected as the representative samples. MatureBays^21^ chooses a ratio of 1 positive to 10 negative samples for each mature miRNA by randomly selecting subset of negative samples. MiRPara^26^ extracts the sequences which have the same strand with true miRNA but randomly shifted by at least 5-bp from the true start position as the negative samples. Although solving the class imbalance problem can improve the accuracy of miRNAs identification, a minority of global methods considered the imbalanced data constitute. The class imbalance problem has been expected to be addressed, and further to improve the performance of mature miRNAs classifier.

To overcome the challenges and to improve the performance of classifier of mature miRNAs, take 5′ mature miRNAs identification as an example, we explored the computational method, namely MatFind, which can be used to identify 5′ mature miRNAs. Firstly, we constructed balance training dataset by clustering and selecting the represent negative samples based on K-nearest neighbor (κ-NN). Secondly, the multiple SVM classifiers were trained in orderly using the balance dataset. Specifically, each next SVM classifier was trained to improve the discriminative ability by reselecting balance train dataset including incorrectly classified samples of the upper classifiers. At last, entire SVM classifiers were combined together to form the ensemble classifier. Thanks for using the balance dataset and classifier ensemble method, our results on independent testing dataset show that the proposed method is more efficient than one without treating class imbalance problem. Moreover, compared with the other mature miRNA identification approaches that are available, MatFind achieves much higher classification accuracy.

## Materials and Methods

### Data

A human pre-miRNAs dataset which is download from the miRBase database (version 18)^30^, is composed of 1220 experimentally verified miRNA precursors. 1120 randomly selecting pre-miRNA sequences of 1220 sequences were used to establish the training dataset. And the remaining 100 sequences were used as the test dataset.

The training dataset was constructed through sliding window technique. In sliding window technique, for each pre-miRNA sequence, a sliding window is used to select all possible sequences which are generated by sliding nucleotides (or 1 base pair) from the first nucleotides of pre-miRNA sequence. Furthermore, of all possible sequences, the sequence which start sit is overlap with the true mature miRNA is used to construct positive dataset, others sequences are used to form the negative dataset.

### Feature set

**Figure 1:**
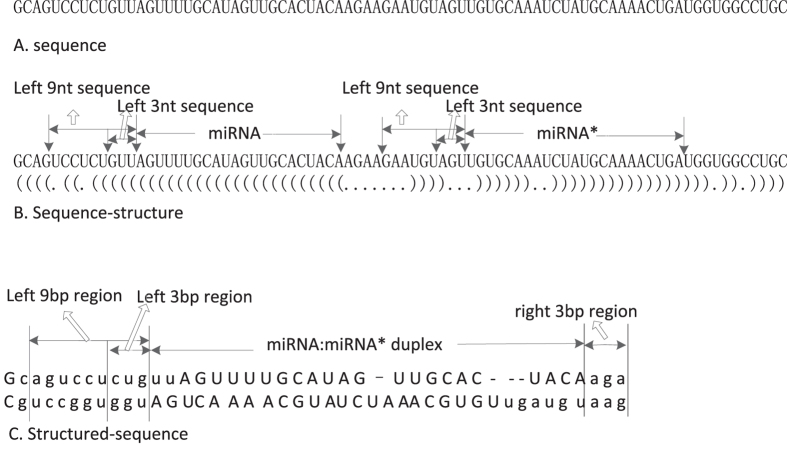
The sequence description for feature exacting (taking has-mir-19a as an example).

In this study, we select the optimal feature set for mature miRNA identification which represents the typical characteristic of mature miRNAs. The optimal feature set can classify the mature miRNA parts and other sequence fragments of their pre-miRNA. Taking has-mir-19a as example, the sequence description for feature exacting is illustrated in Fig. 1. We define the left (or right) ith nt sequence, miRNA and miRNA* in the pre-miRNA sequence. As well as, we define the left (or right) *i-th* nt region and miRNA : miRNA* duplex of the structured per-miRNA sequence.

In our study, the features can be divided into three categories based on exacting sequences: sequence-based features, sequence-structure-based features and structured-sequence-based features. All features were shown in Table 1.

Basis of the specific ontology of the features, the features can be denoted as follows: paired-nucleotides types, combination of nucleotide, length, stability, nucleotide and paired-type and paired states. Of them, paired-nucleotides types can be listed as “NN”, “AA”, “AC”, “AG”, “AU”, “CA”, “CC”, “CG”, “CU”, “GA”, “GC”, “GG”, “GU”, “UA”, “UC”, “UG”, “UU”, “-A”, “-C”, “-G”, “-U”, “A-”, “C-”, “G-”, “U-” and “- -”. In the feature set, they are converted to 0, 1, 2, 3……24 and 25 based on a strict one-to-one correlation. And combination of nucleotide and paired-type include “N.”, “A (”, “C (”, “G (”, “U (”, “A.”, “C.”, “G.”, “U.” and “-.”, of them, “(“or”)” and “ . ” represent pairing and no pairing, respectively. They are converted to 0, 1, 2, 3……8 and 9 based on a strict one-to-one correlation. In addition, paired states can be denoted as 0 (paired) and 1(no paired).

The secondary structure of all sequences was predicted using RNAfold of ViennaRNA-1.8.5^31^ and Perl scripts.

### Classifier construction

#### Data processing

The negative dataset *S*_*neg*_ is special because each negative sample was extracted from their pre-miRNA by sliding 1 base pair (bp) distance. There are similarities of some negative samples in the field of feature composition. Thus, we employ κ-NN^32^ approach to classify all the negative data. The distance of each negative sample and its center of mass was calculated as:


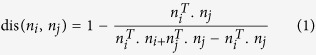


Here, a negative sample was denoted by a feature vector matrix. Let *n*_*i*_ and *n*_*j*_ are the feature vectors of *i-th* and *j-th* negative samples.

**Figure 2:**
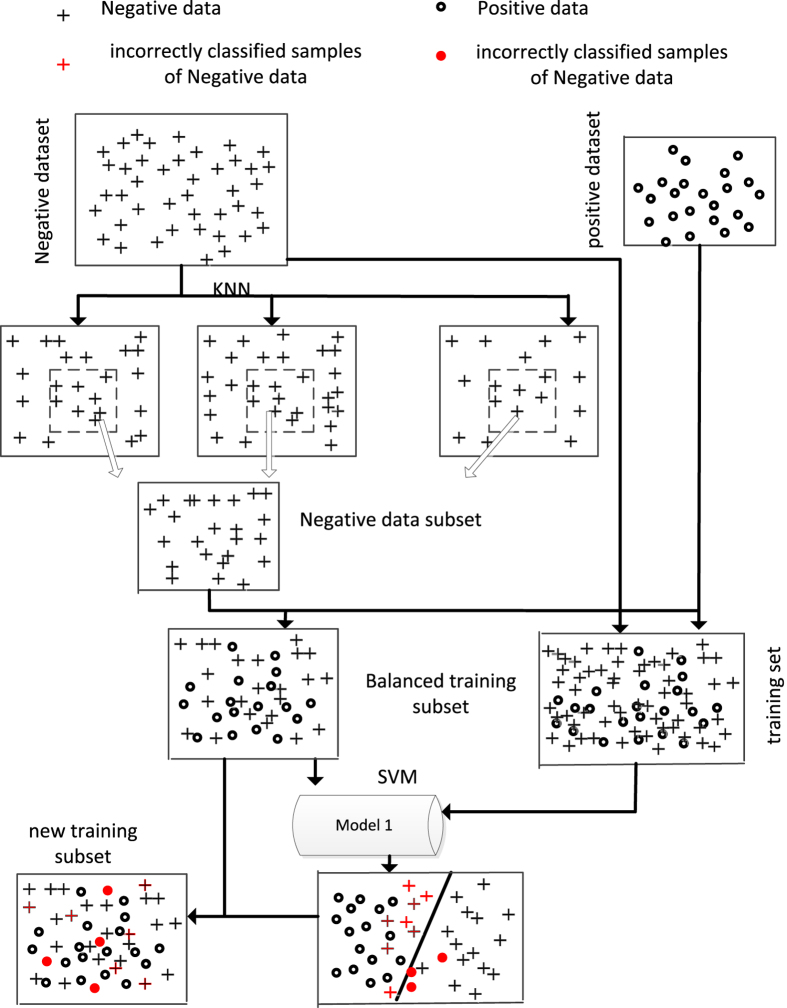


In order to improving the performance of classifier, we strengthen the training subset by combining incorrectly classified samples which are predicted results of prior classifier. The method of training subset selection is illustrated in Fig. 2.

The negative dataset were clustered into 10 groups. Due to the radio of positive and negative is about 1:10, for each group, one-tenth negative samples which have the minimum distance away from the center of mass within each cluster was extracted to form the dataset *S*_*neg*1_. Then, the negative data subset *S*_*neg*1_ was combined with all the positive data *S*_*pos*_ to form the balanced training dataset *S*_*classifier*1_ which is balanced training subset.


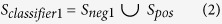


Then, the first mature identification classifier was trained based on SVM. The training dataset *S*_*train*_ was classified by this classifier. Moreover, the incorrectly classified samples *S*_*incor*1_ were selected and combined with *S*_*classifier*1_ to form the new training subset *S*_*classifier*2_ for the next classifier training. Therefore, the data components of training subset *S*_*classifieri*_ can be denote as:


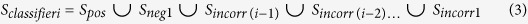


Here, the cluster of negative dataset and distance of each negative sample with its center of mass were calculated using SPASS.

#### SVM classifier

The Support Vector Machine (SVM) has performed excellent classification ability in miRNA identification. Therefore, SVM was adopted to train the weak classifiers. In the case of linear inseparable, for each example *x* to be classified, the classify function can be denoted as:


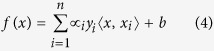


where *x*_*i*_ is a feature vector of sample *x*. And ∝_*i*_ (0 ≤ ∝_*i*_ ≤ *C*) is a coefficient which can be learned, and C is the penalty parameter. In addition, 〈x, x_i_〉 is inner product of x and x_i_. The kernel function is used to calculate the inner product, it solves the linear inseparable problem of the original space through the data mapped to high-dimensional space. Specifically, the Radial Basis Function (RBF) was used as the Kernel function which denoted as:


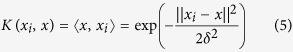


where δ is a regulate control parameter, and determine the feature weight.

**Figure 3:**
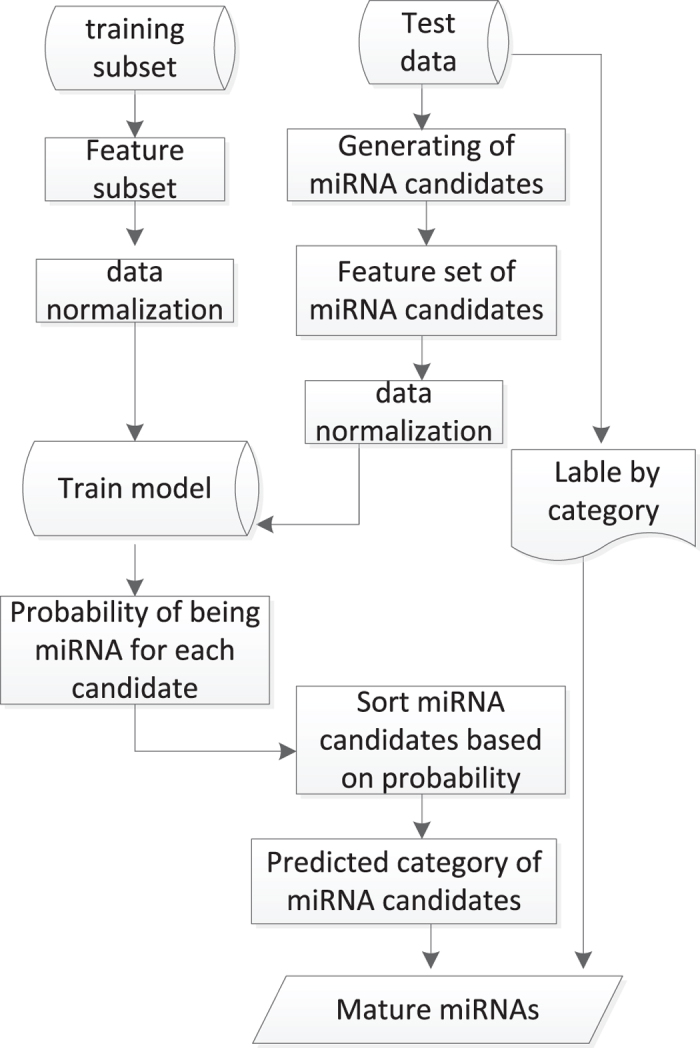
The computational procedure of SVM classifier.

The computational procedure of SVM classifier for mature miRNA classification was illustrated in Fig. 3.

The training process started from exacting feature vector matrix of balanced training subset. In addition to data normalization, we train the miRNA identification model using SVM algorithm. In test process, each sequence of trainning dataset was used to generate multiple sequences which include difference mature miRNA candidates. Then, the feature set vector was extracted from these sequences. By testing with this model, all candidates of each test data sequence were sorted based on probability of being miRNA. The highest probability candidate was considered as true mature miRNA. Specifically, SVM algorithm was implemented using libsvm3.20^33^ and Perl scripts.

#### Ensemble classifier construction

The ensemble method follows the same idea as the Adaboost algorithm, and it is defined as follow:

Input:

Sequence of N examples (*x*_1_, *y*_1_), …., (*x*_*m*_, *y*_*m*_) with labels *y*_*i*_ of test dataset

Initialize D_1_ = (w_11_, w_12_, …w_1i_ …, w_1N_), D_1_(i) = 1/N for all i

For t = 1, …, M, M = 10

Call SVM classifier, providing it with the distribution D_m_

Get the basic classifier G_m_(x): x → {−1, +1}

Test with test dataset

Get back a hypothesis h_m_: X → Y

Error: 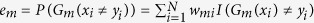


If 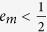, set M = m − 1 and abort loop

Set 
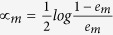


Update


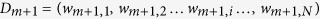
, 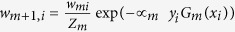, 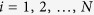


Where *Z*_*m*_ is normalization constant 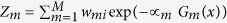


Output: 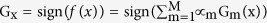.

In the training process, *C* is related with the selection of optimal hyperplane of SVM classifier. e_m_ represents the sum of weight of all incorrectly classified samples. And ∝_m_ is the weight of each classifier in the ensemble classifier. Duo to each classifier was trained using the incorrectly classified samples of prior classifiers, it can classify the samples which can’t be predicted by prior classifiers.

#### Classification of high confidence miRNA candidates

The method of obtaining the high confidence miRNA candidates can be described as follow:: Firstly, each sample of test data was used to generate multiple pre-miRNA sequences which have different start sits of mature miRNAs. Secondly, on basis of each SVM classifier, we obtained the probability that mature miRNA of pre-miRNA is the actual mature miRNA. Then the pre-miRNAs were ordered based on the probability. At last, the candidates of ensemble classifier were obtained based on the sum of different weighted probability. The method enhances the reliability of decision making.

#### Classifier performance estimation

We define the percentage of identified true miRNA relative to the total number of miRNA candidates as prediction accuracy. Assume that there are N miRNA candidates, for *i*–*th* candidate, *S*(*i*) is the number of identified true miRNA, and *N*(*i*) is the total number of miRNA candidates. The accuracy rate of prediction Acc(x) is defined as:


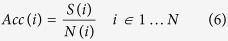


In addition, we define the absolute value of the number of nucleotides between the start sit of predicted mature miRNA and the start sit of true miRNA as position deviation (PD). Whether the start sit of predicted mature miRNA is in front of or behind the true start sit of mature miRNA, the PD is higher than 0. Assume that there are N miRNA candidates, for i–th candidate. The average PD (APD) is defined as:


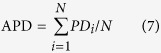


## Results and Discussions

### Dataset of multiple SVM classifiers

The training data included 1118 positive examples and 18098 negative examples. Except the training subset of the first SVM classifier, the training subsets of others SVM classifiers were constructed using the training subset and the incorrectly classified samples of prior classifier. Specifically, the multi-duplicated samples of the training subset were removed. The non-redundancy training subset was utilized for the next model training. The training data component of 10 classifiers was shown in Table 2.

Duo to the feature set captured the typical characters of mature miRNAs, the first classifier which was trained using reduced training subset achieved higher accuracy rate. And attributing to the strengthened data distribution of the incorrectly classified samples, other classifiers also had an excellent performance. As results, their incorrectly classified samples were few. Comparing with the initial training dataset, the positive data and negative data of others training subset were remain balanced.

### Multiple SVM classifiers training

Each SVM classifier was trained based on strengthened data distribution of the incorrectly classified samples. The parameters including error rate (*e*_*m*_), weight of classifier (∝_*m*_) and penalty parameter *C* of SVM training process were closely related to the accuracy rate of classifier. The parameters of 10 classifiers were shown in Table 3.

The penalty parameter *C* and *g* were optimizing selected to confirm the optimal hyperplane. Therefore, they play important roles in model training. For example, in training process of classifier1, if set c = 2048 and g = 0.001953125, the accuracy rate of the first candidate which predicted based on classifier1 achieved 67.12%, but if set c = 2 and g = 0.5 the accuracy rate of the first candidate which predicted based on classifier1achieved 87.07%.

**Figure 4:**
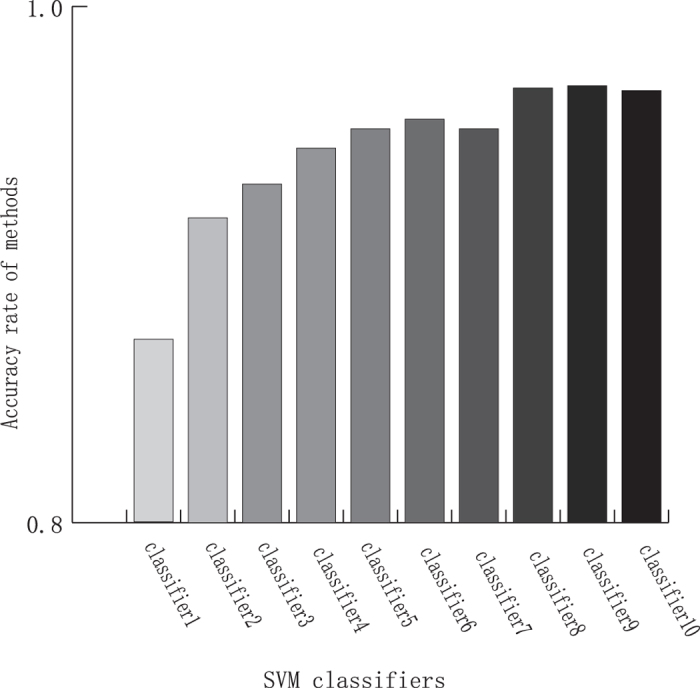
The accuracy rates of the multiple SVM classifiers over the training data.

In some cases, the accuracy rate of classifiers is little descending. This phenomenon attributed to the different classifier has different optimal hyperplane. In addition, some samples were incorrectly classified with many times, so the weights of these samples were increasingly higher, it leaded to the higher error rate. But by and large the accuracy rate of classifiers tends to rise. The Acc of the multiple SVM classifiers over the training data are shown in Fig. 4. And the Acc of the multiple SVM classifiers over the training data based on different position deviation distributions are detail shown in Table 4.

The result was shown that the Acc of then classifiers were also increasing within 5 nt position deviation. It improved 9.67% and 1.02% from classifier 1 to classifier10.

### Comparison of the balanced-data-based and imbalanced-data-based methods

**Figure 5:**
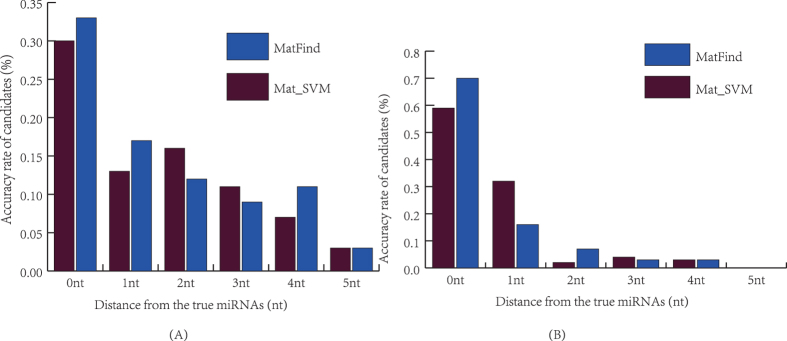
The accuracy rate of the balanced-data-based and imbalanced-data-based methods over test dataset.

In order to compare the performance of the balanced-data-based and imbalanced-data-based methods, we designed the imbalanced-data-based method, namely Mat_SVM, which was trained using all train dataset and design based on SVM. The first candidate’s Acc of the balanced-data-based and imbalanced-data-based methods over test dataset is shown in Fig. 5(A) and detail in Table 5. And the top 5 candidate’s Acc of the balanced-data-based and imbalanced-data-based methods over test dataset is shown in Fig. 5(B) and detail in Table 6.

It can be seen that MatFind achieved excellent prediction accuracy for identifying mature miRNAs. Specifically, the Acc of the first candidates improved 3 nt than Mat_SVM. And the Acc of the top 5 candidates significantly improved 11 nt. In addition, the first candidate can be completely predicted within 5 nt position deviation. Moreover, the APD of the imbalanced-data-based method was 2.19 nt, lower than 2.05 by MatFind.

### Comparison with others methods

**Figure 6:**
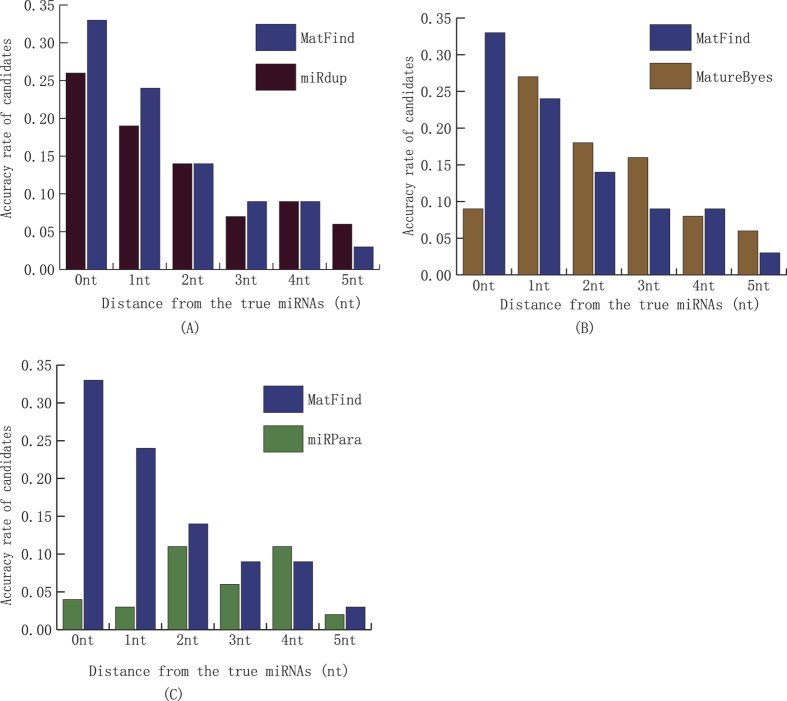
The prediction results of miRdup, MatureByes, MiRPara and MatFind.

Three state-of-the-art methods MatureByes, miRPara, miRdup are publicly available and actively maintained were selected to compare with the proposed MatFind using a set of test data. MatureByes was one of the earliest methods which is develop to predict the mature miRNAs. It found some new features of mature miRNAs which are typical represents for reliably distinguishing between true and pseudo mature miRNAs. MiRPara is a software tools for predicting the most probable mature miRNA from genome scale sequences based on SVM. MiRdup is a computational predictor for the most likely mature miRNAs of their pre-miRNA based on random forest algorithm. The examined ability of them was evaluated by the accuracy rate of these methods for the first candidate with different position deviation. The prediction results are summarized in Fig. 6 and detail in Supplementary Tables 1–3 in Supplementary Material.

As shown in Fig. 6. MatFind significantly outperformed the other methods. MatFind predicted that 33% of the true mature miRNAs, which was significantly better than the 9%, 4% and 26% by MatureByes^21^, miRPara^26^ and miRdup^24^. In addition, MatFind achieved hundred-percent predicted the true mature miRNAs with 5 nt position deviation, significantly higher accuracy rates than 84%, 37% and 81% by Mature Byes, miRPara and miRdup. And the APD of the MatFind was 2.05 nt, the corresponding values for MatureByes, miRPara and miRdup was 4.65 nt, 5.43 nt and 2.67 nt. The results indicated that MatFind achieved more accurate predicted mature miRNA candidates.

## Discussions

The problem of mature miRNA prediction is identification of the true miRNAs from their pre-miRNA. Therefore, the length of pre-miRNA decided the number of mature miRNA candidates. As results, mature miRNA identification is a class imbalance problem. We extracted a balanced dataset as the initial train subset by κ-NN algorithm for the first SVM classifier training. In addition, training subsets which combined incorrectly classify samples were used to train multiple SVM classifiers. This method not only solved the class imbalanced problem, but also improved the performance of classifier for mature miRNA prediction. As evaluatd by independent dataset, our method obtained higher accuracy rate than imbalanced-data-based method. And, more remarkable, the Acc of the first candidates improved 3 nt than Mat_SVM. And the Acc of the top 5 candidates significantly improved 11 nt. Compared with other three methods by independent dataset, our classifier achieved significant improvement on the prediction accuracy rate. Specifically, MatFind predicted that 33% of the true mature miRNAs, which is significantly better than the 9%, 4% and 26% by MatureByes^21^, miRPara^26^ and miRdup^24^. And 100% of the predicted positions were within 5 nt position deviation of mature miRNA start sites. Moreover, our classifier had the smallest APD than other three methods. Therefore, it is shown that our method improved overall performance of mature miRNA prediction.

It should be noted that our method is a classifer for start sit of 5′ mature miRNA prediction. This method does not consider the other sits of mature miRNA, which are also important for mature miRNA. This may further study on constructing models for other sits of mature miRNA identification using our method.

In this work, the training dataset includes 20,000 miRNA:miRNA* duplex sequences and 110 features, our classifier can be constructed within five minutes. Moreover, our classifier takes just a few second to predicted mature miRNAs of one hundred pre-miRNAs in test datset. Therefore, our method has low time complexity. But although our classifier could work with more features or next-generation sequencing, no doubt it is time-consuming operation. As shown in Zou *et al*.^34^, the open source Apache Hadoop project, which work based on the MapReduce frame work and a distributed file system, provide an opportunity to large-scale data processing as well as achieve scalable, efficient and reliable computing preformance for our classifier.

## Conclusion

Although several methods have been developed for mature miRNA identification, the accurate rate of identifying the start sit of mature miRNA from a given pre-miRNA is remain more lower. The mature miRNA prediction is a class-imbalanced problem which also leads to the unsatisfactory performance of these methods.

MatFind utilized balanced training data to train multiple classifiers based on represented features and SVM algorithm. Then integrated them based on the the same idea as the Adaboost algorithm. MatFind achieved a higher prediction improvement than the method which adopted the imbalanced training data for model training. Especially, it has higher prediction performance than other three methods. As results, MatFind is an accurate and fast method for 5′ mature miRNA identification. The method which is on basis of adaboost-SVM algorithm can solve the class-imbalanced problem, as well as achieve a higher performance for method of mature miRNA identification.

There are a serial of measures can be taken to improve our mehtod. As described in iMiRNA-SSF^35^, combining negative sets with different distributions can improving the identification of pre-miRNA, the same methods are also applied in mature miRNA identification. And the LibD3C provided a hybrid model of ensemble pruning that is based on K-means clustering and the framework of dynamic selection and circulating in combination with a sequencital search method^36^. we shall make efforts in our future work to try these methods for improving preformance of our classifier. And as shown in a series of recent publications (see, e.g., iEnhancer-2L^37^ and two studies of Liu *et al*.^38^,^39^), user-friendly and publicly accessible web-servers with more information, such as SVM score,and visulization functions, can significantly enhance their impacts, we shall make efforts in our future work to provide a web-server to displaying findings that can be manipulated by users according to their need.

## Additional Information

**How to cite this article**: Wang, Y. *et al*. Improving classification of mature microRNA by solving class imbalance problem. *Sci. Rep.* **6**, 25941; doi: 10.1038/srep25941 (2016).

## Supplementary Material

Supplementary Information

## Figures and Tables

**Table 1:** All features used by MatFind.

Features	Sequence	_Number_	_Explanation_
pn1–25	structured-sequence	25	paired-nucleotides type of each position of duplex
ss1–50	sequence-structure	50	Combination of nucleotide and paired-type of each position of duplex
fl1–18	sequence-structure	18	Combination of nucleotide and paired-type of each position of left 9 bp region of duplex
fr1–3	structured-sequence	6	Combination of nucleotide and paired-type of each position of right 3 bp region of duplex
MFE1–5	structured-sequence	5	minimum free energy of duplex; minimum free energy of left 3 bp double-stranded sequence; minimum free energy of left 5 bp double-stranded sequence; minimum free energy of left 9 bp double-stranded sequence; minimum free energy of right 3 bp double-stranded sequence
length	structured-sequence	1	Distance from the first position to terminal loop
Num1–3	structured-sequence	3	The number of “−” in double-strand from +2 bp to +5 bp of duplex; The number of “−” in double-strand from +3 bp to +8 bp of duplex; The number of “−” in double-strand from +9 bp to +12 bp of duplex;
fn	sequence	1	The first nucleotide of mature miRNA
pair	structured-sequence	1	paired state of the first base pair of duplex

**Table 2:** The training data component of 10 classifiers.

Classifier	1	2	3	4	5	6	7	8	9	10
Negative Samples(N)	1119	1230	1335	1507	1564	1611	1654	1699	1723	1739
Incorrectly classified Samples(N)	248	146	118	88	72	64	72	38	36	40
Training subset(N)	2237	2348	2453	2625	2682	2729	2772	2817	2841	2857

**Table 3:** The parameters of 10 classifiers.

Classifier	*e*_*m*_	∝_*m*_	g	c	_Acc_
classifier 1	0.013	2.1605	0.5	2	87.07
classifier 2	0.1193	0.9996	0.5	8	91.81
classifier 3	0.3533	0.3023	0.5	8	93.12
classifier 4	0.3611	0.2853	0.5	2	94.51
classifier 5	0.3513	0.3067	0.5	8	95.26
classifier 6	0.3878	0.2283	0.5	2	95.63
classifier 7	0.4169	0.1677	0.5	8	95.26
classifier 8	0.1613	0.8241	0.5	2	96.84
classifier 9	0.4542	0.0917	0.5	2	96.93
classifier 10	0.4337	0.1334	0.5	8	96.74

**Table 4:** The Acc of the multiple SVM classifiers over the training data based on different position deviation distributions.

Classifier	0 nt	1 nt	2 nt	3 nt	4 nt	5 nt
classifier 1	0.8707	0.8986	0.9293	0.9516	0.9516	0.9647
classifier 2	0.9181	0.9451	0.9563	0.9665	0.9665	0.9721
classifier 3	0.9312	0.9386	0.9460	0.9609	0.9609	0.9684
classifier 4	0.9451	0.9479	0.9544	0.9563	0.9563	0.9674
classifier 5	0.9526	0.9544	0.9600	0.9637	0.9637	0.9684
classifier 6	0.9563	0.9628	0.9647	0.9665	0.9665	0.9730
classifier 7	0.9526	0.9535	0.9553	0.9581	0.9581	0.9665
classifier 8	0.9684	0.9693	0.9702	0.9721	0.9721	0.9758
classifier 9	0.9693	0.9712	0.9721	0.9721	0.9721	0.9740
classifier 10	0.9674	0.9684	0.9684	0.9693	0.9693	0.9749

**Table 5:** The first candidate’s accuracy rate of the balanced-data-based and imbalanced-data-based methods over test dataset.

Classifier	0 nt	1 nt	2 nt	3 nt	4 nt	5 nt	Sum
Mat_SVM(%)	0.30	0.23	0.16	0.11	0.07	0.03	1
MatFind(%)	0.33	0.24	0.14	0.09	0.09	0.03	1

**Table 6:** The top 5 candidate’s accuracy rate of the balanced-data-based and imbalanced-data-based methods over test dataset.

Classifier	0 nt	1 nt	2 nt	3 nt	4 nt	5 nt	Sum
Mat_SVM (%)	0.59	0.32	0.02	0.04	0.03	0	1
MatFind(%)	0.70	0.17	0.07	0.03	0.03	0	1
