# Supplementary material for: Improving classification of mature microRNA by solving class imbalance problem
Source: Sci Rep. 2016 May 16;6:25941. doi: 10.1038/srep25941 (PMC4867574; doi:10.1038/srep25941)
Supplement: Supplementary Information [file srep25941-s1.pdf]

Supplementary Information

Improving classification of mature microRNA by solving class imbalance problem

Ying Wang[1,2], Xiaoye Li[1], Bairui Tao[1]

[1]Modern Educational Technology Center, Qiqihar University, No.42, Wenhua Street, Qiqihar, Heilongjiang, China, 161006

[2]Institute of Biomedical Engineering, College of Automation, Harbin Engineering University, 145 Nantong Street, Nangang District, Harbin, Heilongjiang, China, 150001

* Correspondence to: Ying Wang Email: wangying0129@126.com

Modern Educational Technology Center, Qiqihar University, No.42, Wenhua Street, Qiqihar, Heilongjiang, China, 161006

Tel number: 08604522738151

Fax number: 08604522738151

Supplementary Table 1. The accuracy rate of miRdup and MatFind with different position deviation

Classifier	0nt	1nt	2nt	3nt	4nt	5nt	sum
miRdup(%)	0.26	0.19	0.14	0.07	0.09	0.06	0.81
MatFind(%)	0.33	0.24	0.14	0.09	0.09	0.03	1

Supplementary Table 2. The accuracy rate of MatureByes and MatFind with different position deviation

Classifier	0nt	1nt	2nt	3nt	4nt	5nt	sum
MatureByes (%)	0.09	0.27	0.18	0.16	0.08	0.06	0.84
MatFind(%)	0.33	0.24	0.14	0.09	0.09	0.03	1

Supplementary Table 3. The accuracy rate of miRPara and MatFind with different position deviation

Classifier	0nt	1nt	2nt	3nt	4nt	5nt	sum
MiRPara (%)	0.04	0.03	0.11	0.06	0.11	0.02	0.37
MatFind(%)	0.33	0.24	0.14	0.09	0.09	0.03	1
